# PEOS-Net: a Prototype-Energy Open-Set Network for robust tomato leaf disease recognition beyond the closed-world assumption

**DOI:** 10.3389/frai.2026.1938398

**Published:** 2026-10-30

**Authors:** E. Jansi, Kavitha B. R.

**Affiliations:** School of Computer Science Engineering and Information Systems (SCORE), Vellore Institute of Technology, Vellore, Tamilnadu, India

**Keywords:** energy based analysis, open set classification, plant disease, prototype learning, tomato leaf disease

## Abstract

**Introduction:**

Deep convolutional networks now reach high accuracy on curated plant disease benchmarks such as PlantVillage, but nearly all reported systems assume a closed world in which every test image belongs to a known class. In the field, a diagnostic system will meet symptoms, crops, backgrounds or damage types absent from its training taxonomy, and a closed-set classifier will confidently mislabel them, undermining trust in automated crop-advisory tools.

**Methods:**

We introduce the Prototype-Energy Open-Set Network (PEOS-Net), built on a frozen EfficientNet-B0 backbone with a Convolutional Block Attention Module (CBAM) and two parameter-distinct heads that share this representation: an exponential moving average (EMA) prototype memory that drives classification, and a classifier-head branch that yields a free-energy out-of-distribution score. The two scores are fused into one hybrid open-set score; the normalization ranges, fusion weight and rejection threshold are selected on a held-out calibration split and never on the final test partition. We evaluated on an eight-class tomato subset of PlantVillage with two disease classes held out as unknowns, and compared against maximum softmax probability (MSP), out-of-distribution detector for neural networks (ODIN) and OpenMax baselines sharing the same backbone.

**Results:**

PEOS-Net reached 93.87% closed-set accuracy (weighted F1 0.9392). Over five random seeds it achieved an area under the receiver operating characteristic curve (AUROC) of 0.8695 ± 0.0202, an area under the precision-recall curve (AUPR) of 0.8723 ± 0.0192, an open-set classification rate (OSCR) of 0.8363 ± 0.0142 and a false-positive rate at 95% true-positive rate (FPR@95%TPR) of 0.4853 ± 0.0645 (mean ± SD). It outperformed all three baselines on every open-set metric, although the baselines had higher closed-set accuracy (96.5%). In a same-model ablation the hybrid score (AUROC 0.8610) exceeded the distance-only (0.7208) and energy-only (0.8413) scores; a paired bootstrap gave a gain over energy-only of +0.0198 (95% CI 0.0134–0.0262).

**Discussion:**

Weak correlation between the two scores (|r| < 0.06) and low overlap of their errors indicate that they carry complementary information. Because the unknown classes come from the same PlantVillage source, the results support a controlled open-set study rather than validated field deployment.

## 1 Introduction

Timely and accurate diagnosis of crop disease is central to food security. Tomato is one of the most widely cultivated vegetable crops worldwide and is affected by a broad range of fungal, bacterial and pest-related leaf diseases that can be visually distinguished from leaf imagery. Convolutional neural networks trained on curated benchmarks such as PlantVillage now routinely report closed-set classification accuracies above 95% (Mohanty et al., 2016) and efficient backbones such as the EfficientNet family (Tan and Le, 2019) make such models practical to deploy on modest hardware, including field-facing mobile and edge devices. This progress builds on a broader lineage of backbone research, from early deep Convolutional neural networks (CNNs) (Krizhevsky et al., 2012) through deeper architectures (Simonyan and Zisserman, 2015) to vision transformers (Dosovitskiy et al., 2021), virtually all pretrained on large-scale natural-image corpora (Russakovsky et al., 2015) before transfer to agricultural imagery (Kamilaris and Prenafeta-Boldú, 2018).

Almost all of this reported performance, however, is measured under a closed-world assumption: the evaluation set is guaranteed to contain only images drawn from the same fixed taxonomy of classes seen during training. A deployed diagnostic tool cannot make this guarantee. A farmer photographing a tomato leaf may capture a disease not present in the training set, a different crop entirely, a healthy leaf with unusual lighting, insect damage the model was never shown, or simply a non-leaf background. A softmax classifier has no mechanism to express “none of the above”; it is mathematically forced to distribute probability mass over the known classes and will typically do so with high, spurious confidence (Nguyen et al., 2015), directly harming the trustworthiness of the system it is meant to support. This overconfidence problem has motivated a substantial calibration literature, including simple softmax baselines (Hendrycks and Gimpel, 2016), temperature-scaled and input-perturbed detectors (Liang et al., 2018), distance-based Mahalanobis detectors (Lee et al., 2018), and outlier-exposure training (Hendrycks et al., 2019).

This gap is not merely theoretical. Recent large-scale benchmarking work has shown that the choice of fine-tuning paradigm full fine-tuning, linear probing, or visual-prompt tuning measurably changes how well a plant disease model separates known from unknown categories, independent of raw closed-set accuracy (Dong et al., 2024). Separately, ensemble approaches that combine logit-space and feature-space knowledge from both general-purpose pretrained backbones and domain-fine-tuned models have been shown to substantially improve anomaly rejection in plant disease imagery over any single scoring function (Dong et al., 2025). These two results jointly motivate the present work: they confirm that (i) plant disease OSR is sensitive to architectural and training choices in ways closed-set benchmarks do not reveal, and (ii) combining complementary sources of evidence, provided they are genuinely complementary rather than redundant, is an effective strategy, which is precisely the design problem PEOS-Net addresses.

Open-set recognition (OSR) formalizes this requirement: a system must correctly classify samples from known classes while explicitly rejecting samples that do not belong to any of them (Scheirer et al., 2013). A substantial body of OSR work has emerged in general computer vision, ranging from extreme-value recalibration of softmax outputs (Bendale and Boult, 2016) to free-energy scores computed directly from classifier logits (Liu et al., 2020), and metric/prototype-based approaches that use distance to learned class prototypes as a natural, geometrically interpretable rejection signal, building on prototypical networks (Snell et al., 2017).

More recent OSR work has continued to diversify these families, including reciprocal-point formulations that model the complement of each class in embedding space rather than the class itself (Chen et al., 2021), latent energy-based models operating directly on learned feature manifolds (Bao et al., 2023), and broad surveys consolidating this fast-growing literature into a unified taxonomy of OSR strategies (Barcina-Blanco et al., 2023; Geng et al., 2021).

Comparatively little of this literature has been evaluated on plant disease imagery, despite the practical case for OSR being arguably stronger in agriculture than in generic image recognition: field conditions are inherently open-world, and incorrect but confident diagnoses can have direct economic consequences.

A further, more subtle problem motivates the specific design of this paper. Combining multiple open-set scores is often assumed to help—intuitively, a distance-based score and an energy-based score “see” the input differently and should provide complementary evidence. In an earlier iteration of this system, we found this intuition to be wrong when both scores are derived from the same underlying prototype-distance logits: a raw distance score and an energy score computed from that same distance produced component area under the receiver operating characteristic curve (AUROC) values differing by less than 0.001, i.e., combining them added essentially no information. This paper's architecture directly addresses that pitfall by design, and we treat the diagnosis and correction of this failure mode as a methodological contribution in its own right, not only an implementation detail.

This paper makes the following contributions:

- We present PEOS-Net, an open-set recognition architecture that couples a CBAM-attended (Woo et al., 2018) EfficientNet-B0 (Tan and Le, 2019) feature extractor with two structurally independent branches: a prototype-memory branch (Snell et al., 2017) that drives the classification decision, and a separate learned classifier-head branch that drives an energy-based out-of-distribution signal (Liu et al., 2020) fused into a single hybrid open-set score.
- We empirically demonstrate, via correlation and error-overlap analysis, that the two branches are effectively uncorrelated (Pearson |*r*| < 0.05 for both known and unknown samples) and produce qualitatively different errors–the prototype branch recovers 100% of the energy branch's false rejections on known samples–and verify via paired bootstrap that this complementarity yields a statistically significant improvement in fused open-set AUROC (+0.027, 95% CI excluding zero).
- We evaluate PEOS-Net with a comprehensive open-set protocol AUROC, AUPR, FPR@95%TPR, and Open-Set Classification Rate (OSCR) (Dhamija et al., 2018) together with a full hybrid-weight sensitivity sweep, embedding-space discriminability analysis [t-SNE (van der Maaten and Hinton, 2008), uniform manifold approximation and projection (UMAP) (McInnes et al., 2018), intra-/inter-class distance statistics].
- Applied to an eight-class tomato leaf disease subset of PlantVillage with a 2,000-image open-set test partition, PEOS-Net attains 94.75% closed-set accuracy alongside an open-set AUROC of 0.8835 and OSCR of 0.8524, while remaining lightweight by keeping the backbone frozen.

We note that the present evaluation is a within-dataset open-set study: unknown samples are drawn from the same PlantVillage source, imaging conditions, and crop (tomato) as the known classes, differing only in disease label. This controlled setting isolates the open-set scoring mechanism from confounding distribution shift, but does not by itself establish robustness to the broader field-deployment scenarios (different crops, uncontrolled backgrounds and lighting, non-leaf imagery) that motivate this work; we discuss this limitation further in Section 5.11.

The remainder of the paper is organized as follows. Section 2 reviews related work in plant disease classification and open-set recognition. Section 3 describes the dataset and the PEOS-Net architecture and training procedure in detail, including all governing equations. Section 4 summarizes the experimental setup. Section 5 reports closed-set and open-set results, the component ablation, the sensitivity study, statistical tests, embedding-space analysis, qualitative visualizations, computational efficiency, and a discussion of limitations. The paper closes with a concluding section summarizing contributions and directions for future work.

## 2 Related work

### 2.1 Deep learning for plant disease classification

The PlantVillage dataset (Hughes and Salathé, 2015) catalyzed a wave of deep-learning approaches to plant disease diagnosis; Mohanty et al. (2016) showed that standard CNN architectures fine-tuned on PlantVillage could exceed 99% closed-set accuracy under controlled imaging conditions, and Ferentinos (2018) confirmed comparable accuracy across several CNN architectures on a larger 87,848-image corpus. Too et al. (2019)) systematically compared fine-tuning strategies across architectures, and Kamilaris and Prenafeta-Boldú (2018) and Barbedo (2018) survey the broader use of deep learning in agricultural imaging, both noting that most reported accuracies are measured on laboratory-condition images that do not reflect field deployment. Singh et al. (2020) directly quantified this gap by introducing PlantDoc, a field-condition dataset, showing substantially lower accuracy for models trained only on curated laboratory data, a finding directly relevant to this paper's open-set motivation. Subsequent work has focused on efficiency [e.g., the EfficientNet family (Tan and Le, 2019), which scales depth, width and resolution jointly] and on fine-grained localization via attention modules. The Convolutional Block Attention Module (Woo et al., 2018) sequentially applies channel and spatial attention to intermediate feature maps, building on earlier channel-attention work such as Squeeze-and-Excitation networks (Hu et al., 2018), and has been widely adopted in agricultural vision pipelines to help the network focus on lesion regions rather than background foliage.

Domain-shift-aware approaches have also emerged as a parallel strand relevant to this paper's open-set motivation: unsupervised domain adaptation has been used both for cross-species disease severity transfer and for adapting laboratory-trained models to field conditions without additional annotation (Wu et al., 2023), underscoring that closed-set accuracy alone does not guarantee robustness to the kinds of distributional shift a deployed system will encounter.

### 2.2 Open-set recognition

Scheirer et al. (2013) formalized open-set recognition as a constrained risk minimization problem that explicitly considers *open space risk*, defined as the risk of assigning known-class labels to unbounded and previously unseen regions of the input space. OpenMax (Bendale and Boult, 2016) extended this concept to deep neural networks by replacing the conventional softmax output layer with an extreme-value-theory-based recalibration mechanism using the distance between activation vectors and their corresponding class-wise mean activation vectors, establishing one of the earliest practical deep open-set recognition (OSR) baselines.

More recently, energy-based approaches have demonstrated that the free-energy formulation derived from classifier logits provides a more reliable out-of-distribution (OOD) detection score compared with conventional softmax confidence (Liu et al., 2020). The energy score is defined in Equation 1 as


$$\begin{array}{l} E\left(x\right)=-\log\overset{K}{\underset{k=1}{\sum }}\exp\left(l_{k}\right), \end{array}$$ (1)

where *l*_*k*_ represents the logit corresponding to class *k*. Unlike softmax probabilities, which are affected by normalization-induced overconfidence, energy-based scores provide a more effective measure of model uncertainty. This family of confidence-calibration methods also includes temperature-scaled and gradient-perturbed detectors (Liang et al., 2018), class-conditional Gaussian distance detectors (Lee et al., 2018), and training-time outlier exposure (Hendrycks et al., 2019). Alternatively, metric-learning and prototype-based approaches, introduced through few-shot learning frameworks such as Prototypical Networks (Snell et al., 2017) and grounded in a longer metric-learning lineage (Schroff et al., 2015; Wen et al., 2016), represent each class using a learned embedding-space centroid (prototype).

Beyond these three canonical families, reciprocal-point learning reframes the open-space risk problem by learning, for each known class, a point representing the extra-class (“otherness”) space rather than the class itself, and rejecting inputs that fall closer to their own reciprocal point than to any class prototype (Chen et al., 2021). Latent-space energy-based models extend the free-energy formulation of Liu et al. (2020) from raw logits into a learned feature manifold, improving fine-grained separability between visually similar known and unknown classes (Bao et al., 2023) a setting directly analogous to distinguishing visually similar tomato diseases from unseen ones. A recent comprehensive survey consolidates these and other OSR strategies (discriminative, generative, and distance-based) into a single taxonomy and highlights prototype-based methods as a particularly active sub-area due to the compact, geometrically interpretable class boundaries they induce (Barcina-Blanco et al., 2023), a conclusion echoed by Geng et al. (2021)'s independent survey of the same landscape.

Classification is performed based on the distance between an input embedding and the corresponding class prototype, while the same distance measure can be extended for open-set rejection through an appropriate threshold. Each of these approaches captures a distinct geometric or probabilistic interpretation of unfamiliarity: open-space risk modeling focuses on unknown regions, energy-based methods quantify classifier uncertainty, and prototype-based approaches measure deviation from known class representations. These complementary perspectives motivate the hybrid open-set recognition design explored in this work.

### 2.3 Open-set evaluation protocols

Beyond threshold-independent separability metrics (AUROC, AUPR), Dhamija et al. (2018) introduced the Open-Set Classification Rate (OSCR) curve, which sweeps a rejection threshold and jointly tracks the correct-classification rate on known samples against the false-acceptance rate on unknown samples, summarizing both closed- and open-set behavior in a single curve and its area. We adopt OSCR as the headline open-set metric in this work, alongside AUROC, AUPR and FPR@95%TPR, following standard practice in the OSR literature.

### 2.4 Open-set recognition in agricultural imaging

Compared to the closed-set plant disease literature, open-set treatments remain sparse, even though the deployment argument for OSR is arguably stronger in agriculture: field images are collected under uncontrolled conditions by non-expert users, and a system that silently mislabels an unfamiliar symptom as a known disease can lead to inappropriate treatment.

Where open-set treatments do exist in agricultural imagery, they remain fragmented across problem framings. Fuentes et al. (2021) approached the problem as open-set domain adaptation, detecting unknown disease instances specifically to cope with new field farms and unseen environmental conditions, rather than benchmarking against a held-out set of unknown classes in a fixed dataset. Jiang et al. (2022) evaluated both a two-head out-of-distribution network and a semi-supervised OpenMatch-based OSR model on a strawberry disease dataset within a continual-learning framework, underscoring how inconsistent OSR protocols in this domain still are. Dong et al. (2024) benchmarked multiple *post-hoc* OOD detection methods across fine-tuning paradigms on plant disease imagery, but did not propose a new architecture, focusing instead on evaluating existing scores. Dong et al. (2025) proposed a knowledge-ensemble method fusing general and domain-specific knowledge in logit and feature space, reporting strong gains over single-score baselines across CNN, vision transformers (ViTs) and vision-language backbones, and is, to our knowledge, the closest existing analog to a hybrid-score plant disease OSR system. Dong et al. (2023) further extended this direction with a dynamic, incrementally updated open-world detector that relearns rejected unknown categories once labels become available. None of these works, however, address score redundancy when the fused signals derive from the same underlying representation, nor do they target tomato leaf disease specifically with a lightweight approach. This paper fills the gap of the frozen-backbone deployment constraint.

This paper targets that gap directly, using tomato leaf disease as a concrete, practically motivated case study. Table 1 summarizes representative open-set recognition (OSR) approaches and highlights their respective limitations in the context of agricultural image analysis. Existing methods have primarily focused on either prototype-based representation learning or energy-based uncertainty estimation, while most studies have evaluated these techniques independently or as *post-hoc* scoring mechanisms, without examining whether combined signals are genuinely complementary rather than redundant. The comparison presented in Table 1 identifies these research gaps and motivates the proposed PEOS-Net, which integrates prototype-memory learning with an independent energy-based branch to provide robust and complementary open-set recognition for tomato leaf disease classification.

**Table 1:** Comparison of existing open-set recognition approaches and the contributions addressed by PEOS-Net.

Paper (author, year)	Method used	Limitations	Gap addressed by PEOS-Net
OpenMax (Bendale and Boult, 2016)	Extreme-value-theory recalibration	Relies only on softmax activation statistics.	Jointly trained prototype-memory branch and independent energy branch.
Energy-based OOD (Liu et al., 2020)	Free-energy score computation	Provides only a single scalar uncertainty measure.	Separates the energy branch from the prototype branch for complementary uncertainty estimation.
Prototypical networks (Snell et al., 2017)	Embedding-space centroids and prototype distance	No explicit open-set rejection mechanism.	Uses prototype distance as one branch for open-set recognition.
ARPL (Chen et al., 2021)	Adversarial margin optimization	No redundancy analysis between fused confidence signals; assumes complementary signals without statistical verification.	Validates fusion effectiveness through explicit statistical independence testing (paired significance, *p* < 0.001) between branches.
Two-head network/OpenMatch (Jiang et al., 2022)	OOD two-head network vs. semi-supervised OpenMatch OSR model (strawberry disease)	The two evaluated methods rely on incompatible training assumptions (implicit vs. explicit outlier exposure) and were reported as not directly comparable.	Provides a single unified architecture with a consistent training protocol, avoiding the incomparability problem raised in this study.
Fine-tuning paradigms and OOD detection (Dong et al., 2024)	*Post-hoc* OOD detection methods	No dedicated open-set architecture.	Proposes a fused open-set architecture instead of only *post-hoc* uncertainty estimation.
Knowledge-ensemble anomaly detection (Dong et al., 2025)	Domain-specific knowledge fusion	Requires multiple large backbones.	Uses a single lightweight frozen EfficientNet-B0 backbone with statistically validated branch independence.

## 3 Materials and methods

### 3.1 Dataset

Experiments use an eight-class tomato leaf disease subset drawn from the PlantVillage corpus. The known-class data is organized into three partitions: a training set, a validation set, and a known-class test set. The open-set (“unknown”) data, drawn from two representative disease classes never seen during training, is further split into two disjoint subsets: a calibration-unknown subset and a held-out final-test-unknown subset, each containing 500 images per unknown class. The eight known classes and the two unknown classes are shown together in Figure 1.

**Figure 1:**
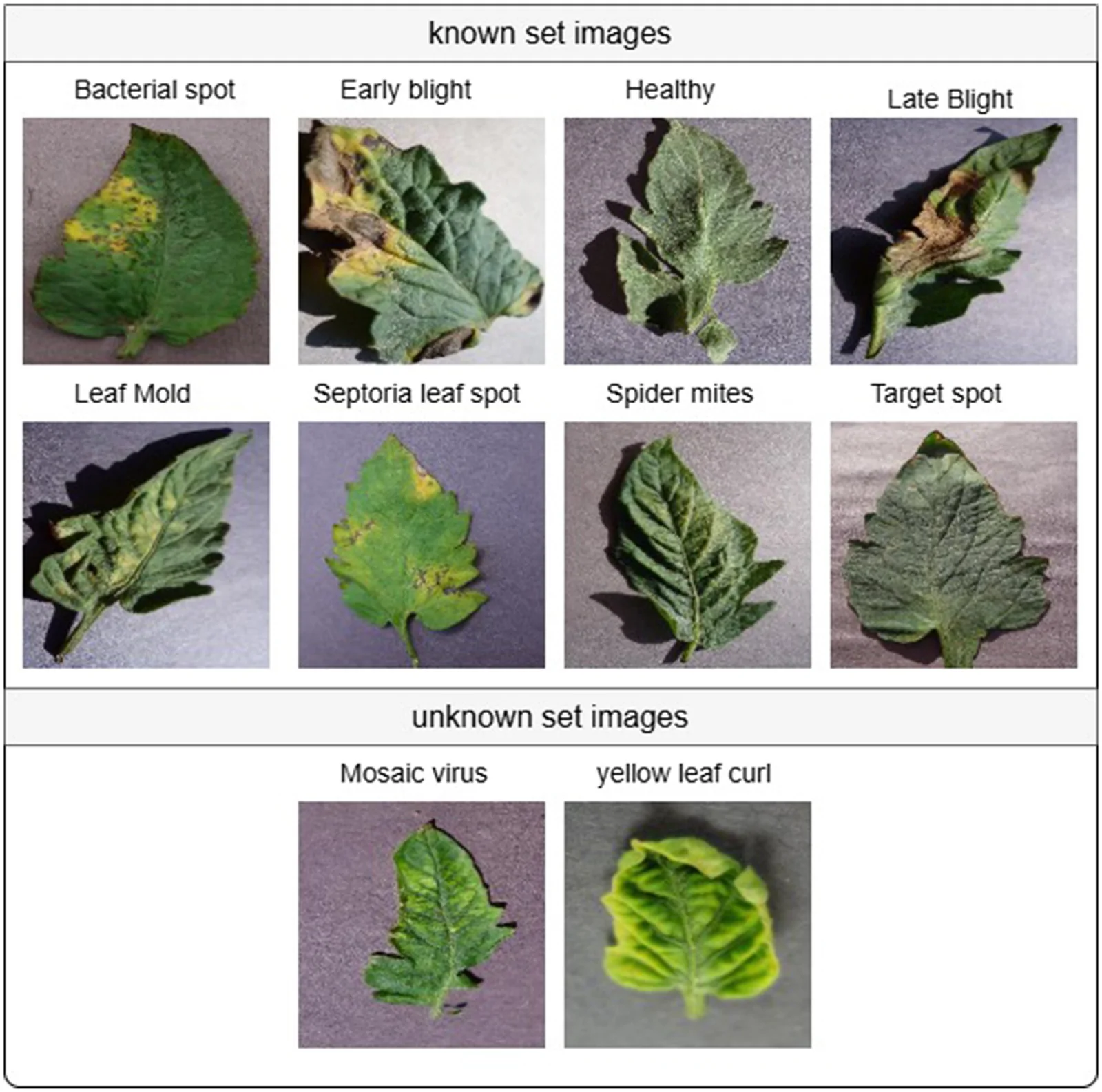
Representative samples from the eight known tomato leaf disease classes used for closed-set training and evaluation, along with two unknown classes reserved exclusively for open-set testing. The unknown classes are excluded from training, validation, and model selection, and are used only to assess out-of-distribution rejection performance. The leaf images are taken from the publicly available PlantVillage dataset.

No data augmentation was applied during training; all images were used as provided by the PlantVillage source subset after resizing and EfficientNet-specific preprocessing mentioned in Table 2. The calibration/final-test unknown split (Section 3.1) was generated once with a fixed random seed (42) and is not redrawn between runs.

**Table 2:** Per-partition image counts.

Partition	Images
Train (known, eight classes)	7,200
Calibration known (val_known_class)	800
Calibration unknown (val_unknown_class)	1,000 (500/class)
Final test known (test_known_class)	800
Final test unknown (test_unknown_class, held-back)	1,000 (500/class)

This yields a three-way experimental protocol—training, calibration, and final test—rather than a simple train/validation/test split, in order to avoid evaluation leakage in the open-set hyperparameters. The *calibration set*, comprising the known validation set and the calibration-unknown subset, is used exclusively to fit the score normalization ranges, select the hybrid fusion weight, and set the rejection threshold (Section 3.7). The *final test partition*, comprising the known-class test set and the held-out final-test-unknown subset, is never used for training or for any hyperparameter selection; it is scored exactly once, after every open-set hyperparameter has already been fixed from the calibration set alone.

All images are resized to 224 × 224 and normalized with the EfficientNet-specific preprocessing function (per-channel scaling matching the ImageNet pretraining statistics).

### 3.2 Problem formulation

Let the training distribution contain samples from *K* known classes, where *y*∈{1, …, *K*}. At test time, inputs may originate either from one of the *K* known classes or from an unknown distribution outside the training taxonomy. The goal of open-set recognition is twofold: (i) for *x* drawn from a known class, predict ŷ = *y* with high accuracy; and (ii) for *x* drawn from the unknown distribution, assign a rejection decision rather than any of the *K* known labels.

We operationalize the rejection decision using a scalar open-set score *S*(*x*), where larger values indicate a higher likelihood that *x* belongs to an unknown class. Given a decision threshold θ, the prediction rule is given by Equation 2.


$$\begin{array}{l} ŷ=\{\begin{array}{cc} \arg\underset{k\in \left\{1,\ldots ,K\right\}}{\max}p\left(y=k|x\right), & \text{if}S\left(x\right)\leq \theta ,\\ \text{Unknown}, & \text{if}S\left(x\right)>\theta . \end{array} \end{array}$$ (2)

### 3.3 PEOS-Net architecture

PEOS-Net (Figure 2) consists of seven stages: (a) Input image, (b) a frozen EfficientNet-B0 backbone, (c) CBAM attention block, (d) shared global-average-pooling bottleneck, (e) two structurally independent heads—a prototype-memory branch that produces the classification decision and the distance-based open-set signal, and an independent classifier-head branch that produces the energy-based open-set signal, (f) the two signals are fused *post hoc* into a single hybrid open-set score, (g) finally open set decision stage. The feature extraction backbone of PEOS-Net is EfficientNet-B0 (Tan and Le, 2019), initialized with ImageNet-pretrained weights. During training, all convolutional layers of the backbone remain frozen, allowing the network to retain rich generic visual representations while substantially reducing the number of trainable parameters. This design keeps PEOS-Net computationally efficient and limits the effective model capacity, thereby mitigating overfitting on the relatively small training dataset containing 7,200 images. Consequently, optimization is restricted to the CBAM attention module, the feature projection layers, and the two task-specific heads, enabling efficient adaptation to the open-set plant disease recognition task.

**Figure 2:**
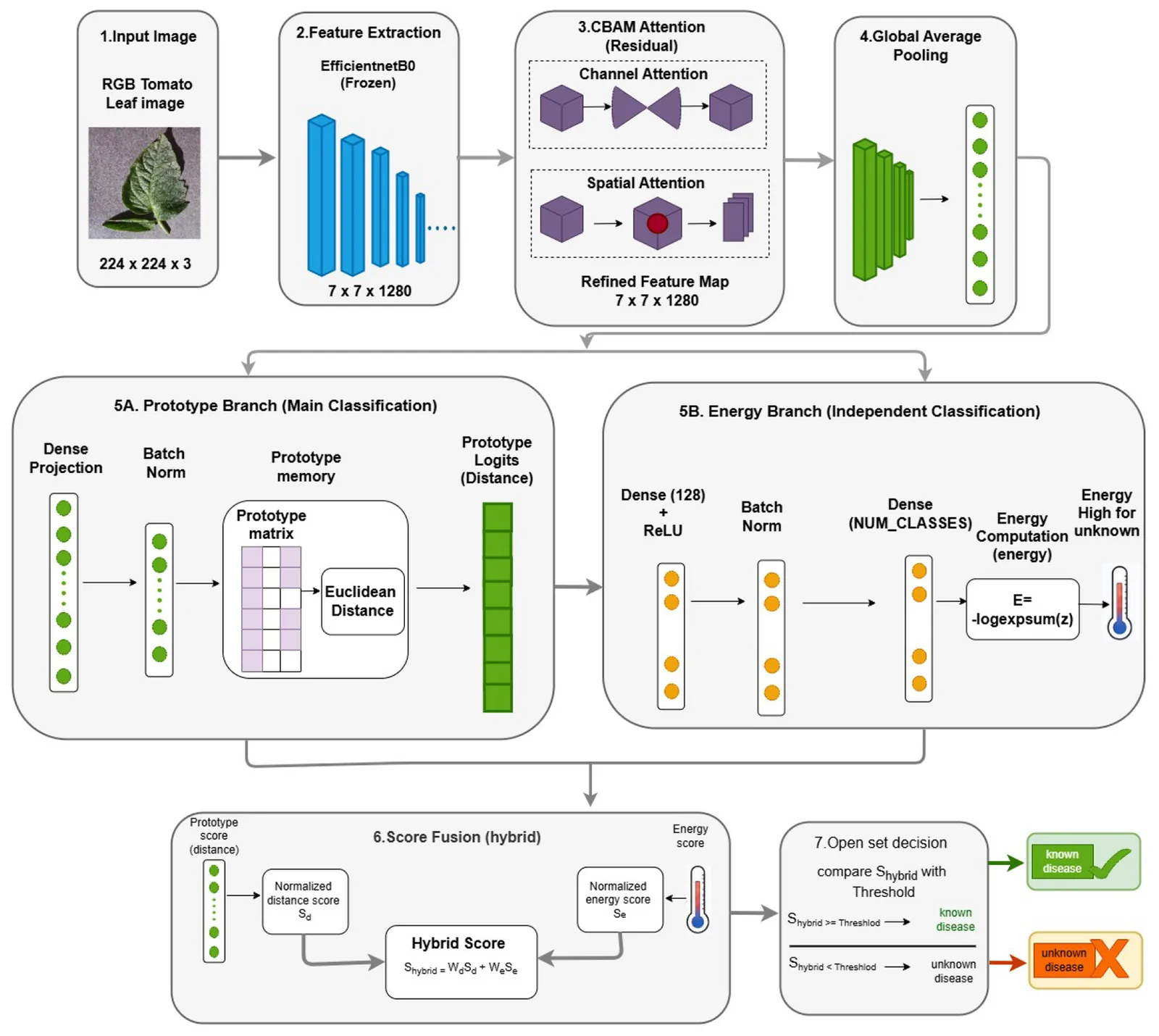
Overall architecture of PEOS-Net.

### 3.4 CBAM attention

Given an intermediate feature map *F*∈ℝ^*H*×*W*×*C*^, channel attention is computed as in Equation 3,


$$\begin{array}{r} M_{c}\left(F\right)=\sigma (\text{BN}(\text{ReLU}(W_{1}\left(W_{0}\left(\text{AvgPool}\left(F\right)\right)\right)+\\ W_{1}(W_{0}(\text{MaxPool}(F)))))), \end{array}$$ (3)

where *W*_0_ and *W*_1_ are the shared-weight dense layers of a channel-reduction MLP (reduction ratio *r* = 8), and the channel-refined feature is


$$F^{\prime }=F⊙M_{c}\left(F\right).$$

Spatial attention is then computed on *F*′ as in Equation 4,


$$\begin{array}{r} M_{s}\left(F^{\prime }\right)=\sigma \left({\text{Conv}}_{7\times 7}\left(\left[{\text{AvgPool}}_{c}\left(F^{\prime }\right);{\text{MaxPool}}_{c}\left(F^{\prime }\right)\right]\right)\right),\\ F^{\prime \prime }=F^{\prime }⊙M_{s}\left(F^{\prime }\right), \end{array}$$ (4)

where AvgPool_*c*_ and MaxPool_*c*_ denote pooling along the channel dimension and [·;·] represents channel-wise concatenation.

The final CBAM output is obtained using a residual connection followed by batch normalization (Equation 5),


$$\begin{array}{l} \text{CBAM}\left(F\right)=\text{BN}\left(F^{\prime \prime }+F\right). \end{array}$$ (5)

This implementation closely follows the original CBAM formulation while incorporating an additional batch-normalization/rectified linear unit (ReLU) gate in the channel-attention branch together with a residual-plus-normalization output stage, which stabilizes optimization when the EfficientNet backbone remains frozen.

### 3.5 Prototype-memory branch (classification)

The shared pooled feature


$$\bar{x}=\text{GAP}\left(\text{CBAM}\left(x\right)\right)$$

is projected into a 256-dimensional embedding


$$f\left(x\right)=\text{BN}\left({\text{Dense}}_{256}\left(\bar{x}\right)\right).$$

PEOS-Net maintains one EMA prototype vector *p*_*c*_ for each known class *c*. The prototype is initialized upon first observation and updated at every training iteration using Equation 6,


$$\begin{array}{l} p_{c}\leftarrow mp_{c}+\left(1-m\right)\cdot \frac{1}{N_{c}}\underset{i:y_{i}=c}{\sum }f\left(x_{i}\right),\;m=0.9, \end{array}$$ (6)

where *N*_*c*_ denotes the number of samples belonging to class *c* in the current mini-batch.

Classification is performed using the squared Euclidean distance in Equation 7,


$$\begin{array}{l} d^{2}\left(x,c\right)=||f\left(x\right)|{|}^{2}-2f{\left(x\right)}^{⊤}p_{c}+||p_{c}|{|}^{2},\;{\text{logits}}_{\text{proto}}{\left(x\right)}_{c}=-d^{2}\left(x,c\right), \end{array}$$ (7)

with predicted label


$$ŷ=\arg\underset{c}{\max}{\text{logits}}_{\text{proto}}{\left(x\right)}_{c}.$$

The prototype-distance open-set score is given by Equation 8.


$$\begin{array}{l} \text{minDist}\left(x\right)=\sqrt{\underset{c}{\min}d^{2}\left(x,c\right)+\varepsilon }. \end{array}$$ (8)

### 3.6 Independent classifier-head branch (energy)

A second classification head receives the same shared feature vector $\bar{x}$ but has completely independent learnable parameters,


$$\text{clsLogits}\left(x\right)={\text{Dense}}_{K}\left(\text{BN}\left(\text{ReLU}\left({\text{Dense}}_{128}\left(\bar{x}\right)\right)\right)\right).$$

Unlike the prototype branch, this classifier contains no shared parameters beyond the common CBAM–GAP feature extractor.

Its open-set confidence measure is computed using the free-energy formulation in Equation 9


$$\begin{array}{l} \text{Energy}\left(x\right)=-\log\left(\overset{K}{\underset{k=1}{\sum }}\exp\left(\text{clsLogits}{\left(x\right)}_{k}\right)\right), \end{array}$$ (9)

where higher energy values indicate greater uncertainty that the sample belongs to any known class. Following Liu et al. (2020), no temperature scaling is applied to the logits prior to this computation (equivalently, *T* = 1); we did not find a temperature-sweep necessary given the calibration-only selection already applied to the fusion weight and threshold.

This independent energy branch was introduced after observing that computing both distance and energy from identical prototype-distance logits produced nearly identical AUROC values, providing little complementary information. Learning the energy score through an independent classifier enables the network to capture a distinct representation of uncertainty while sharing only the common feature extractor.

### 3.7 Hybrid open-set fusion

Both prototype-distance and energy scores are normalized using Min-Max normalization computed exclusively from the *calibration* known distribution (Section 3.1)—never from the final test partition, normalized using min-max normalization (Equation 10),


$$\begin{array}{l} s^{\prime }=\frac{s-lo_{\text{calib}}}{hi_{\text{calib}}-lo_{\text{calib}}}, \end{array}$$ (10)

where *lo*_calib_ and *hi*_calib_ denote the minimum and maximum of each raw score over the calibration-known set, and the same fixed affine transformation is subsequently applied, unchanged, to calibration-unknown, final-test-known, and final-test-unknown samples. Because *lo*_calib_ and *hi*_calib_ are estimated from the calibration-known set alone, a raw score outside this range can, in principle, map to *s*′ < 0 or *s*′>1 on other partitions; we do not clip *s*′ to [0, 1], allowing the hybrid score to extrapolate mildly beyond the calibration range rather than saturating, which we found empirically preserves more separability in the tails of the score distribution than hard clipping. The final hybrid open-set score (Equation 11) is


$$\begin{array}{l} S_{\text{hybrid}}\left(x\right)=w_{d}s_{\text{dist}}^{\prime }\left(x\right)+w_{e}s_{\text{energy}}^{\prime }\left(x\right),\;w_{d}=0.20,\;w_{e}=0.80, \end{array}$$ (11)

The weighting coefficients were selected by maximizing AUROC on the calibration set alone (calibration-known plus calibration-unknown scores) through a reference sweep over


$$w_{d}\in \left\{0.0,0.1,\ldots ,1.0\right\},\;w_{e}=1-w_{d},$$

with the final test partition held out and untouched throughout this selection. The selected weights, along with the normalization ranges (*lo*_calib_, *hi*_calib_) and the rejection threshold θ (Section 3.8), are fixed prior to any final-test evaluation and applied to the final test partition exactly once. Classification labels are always obtained from the prototype branch, while the energy branch contributes exclusively to open-set rejection.

### 3.8 Training and inference procedures

Algorithms 1–3 together define the complete training, calibration, and inference pipeline of PEOS-Net. Algorithm 1 is executed once, during the offline training phase, and jointly optimizes the CBAM-refined backbone, the classifier head, and the prototype memory *P*. At each iteration, the frozen EfficientNet-B0 backbone produces feature maps that are refined by CBAM and pooled into a compact 256-dimensional embedding. From this embedding, the prototype branch computes class-wise squared distances and corresponding logits, while the classifier branch independently produces auxiliary logits used for the energy score. Both branches are optimized jointly through the combined loss *L* = *L*_proto_+λ*L*_cls_, while the prototype memory is updated online through an exponential moving average with momentum *m* = 0.9, allowing class representations to evolve smoothly and stably across training without being dominated by individual mini-batch statistics.

Algorithm 1 Training procedure of PEOS-Net. 
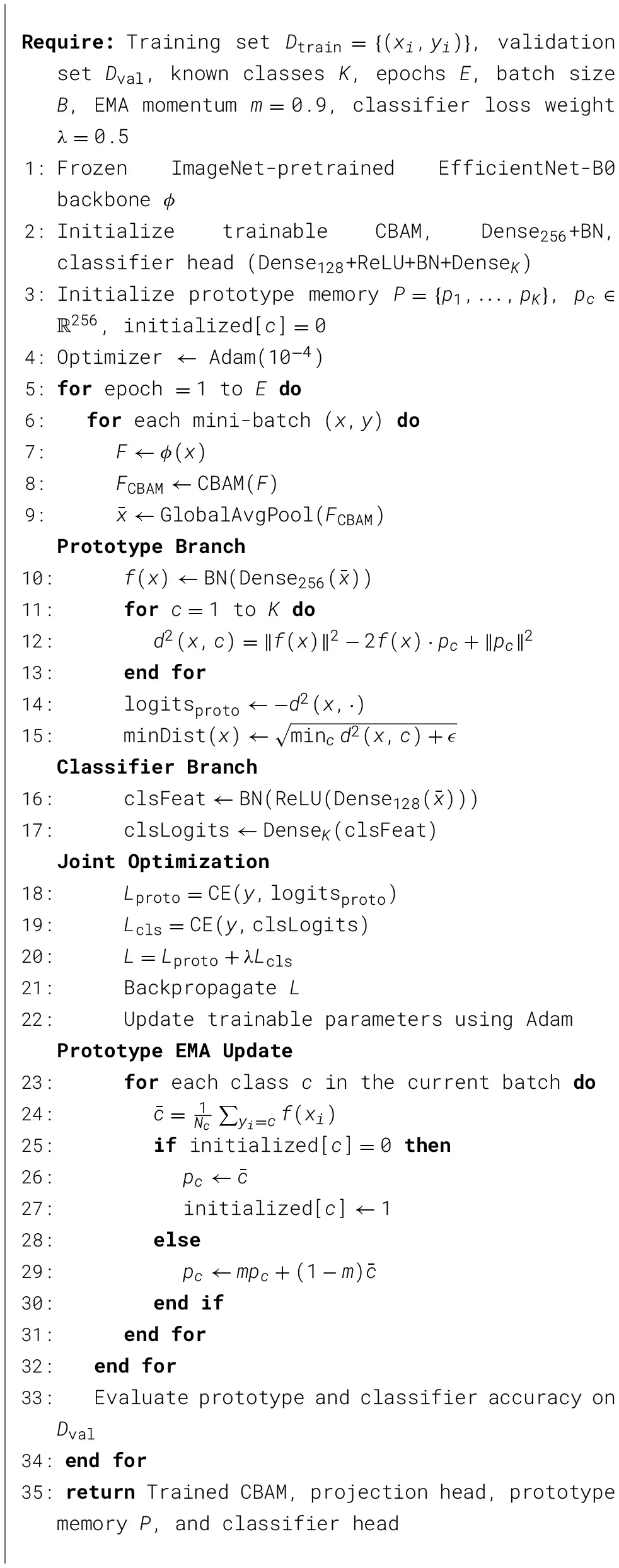

Algorithm 2 PEOS-Net calibration procedure. 
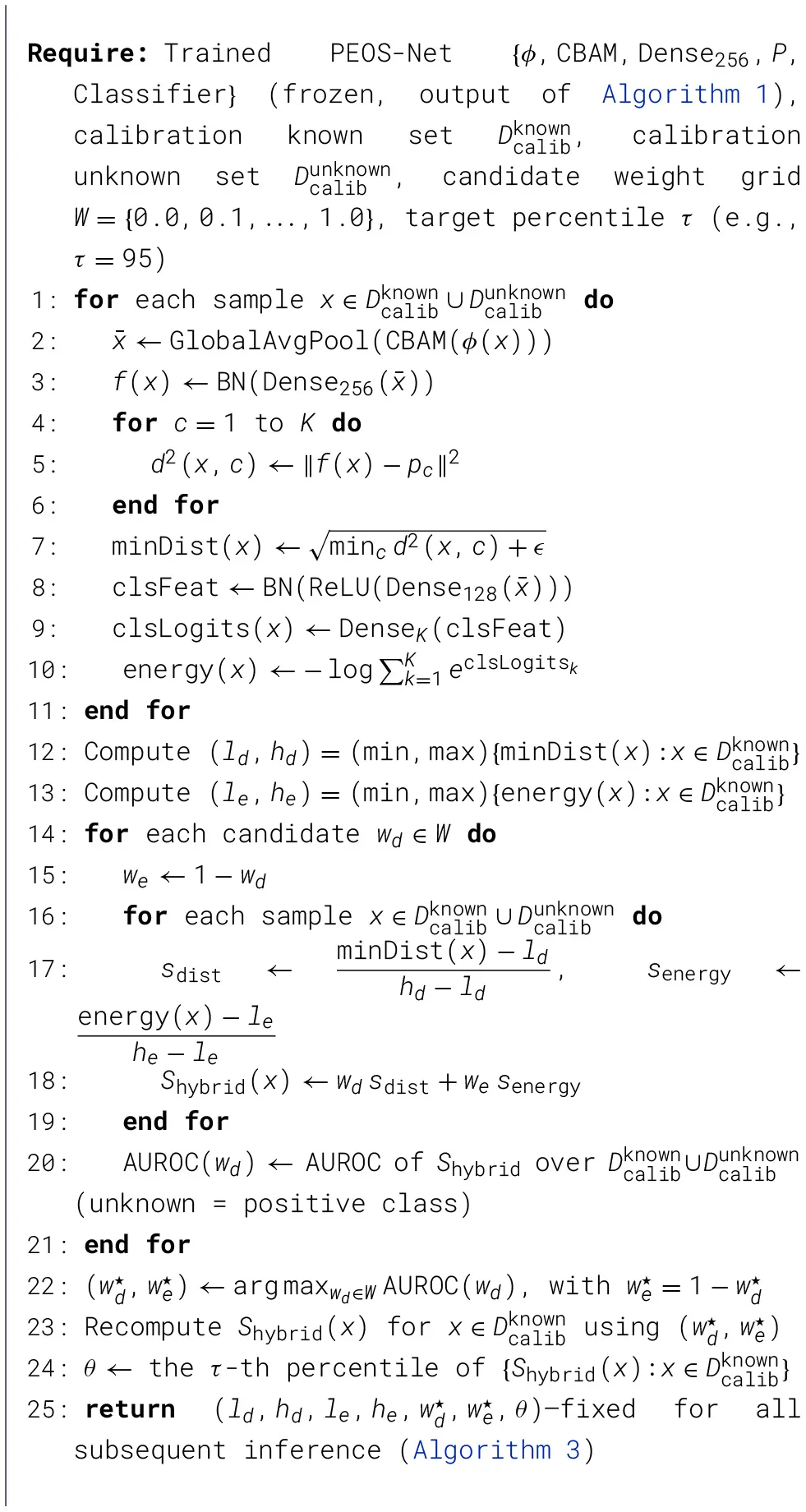

Algorithm 3 PEOS-Net open-set inference and hybrid scoring. 
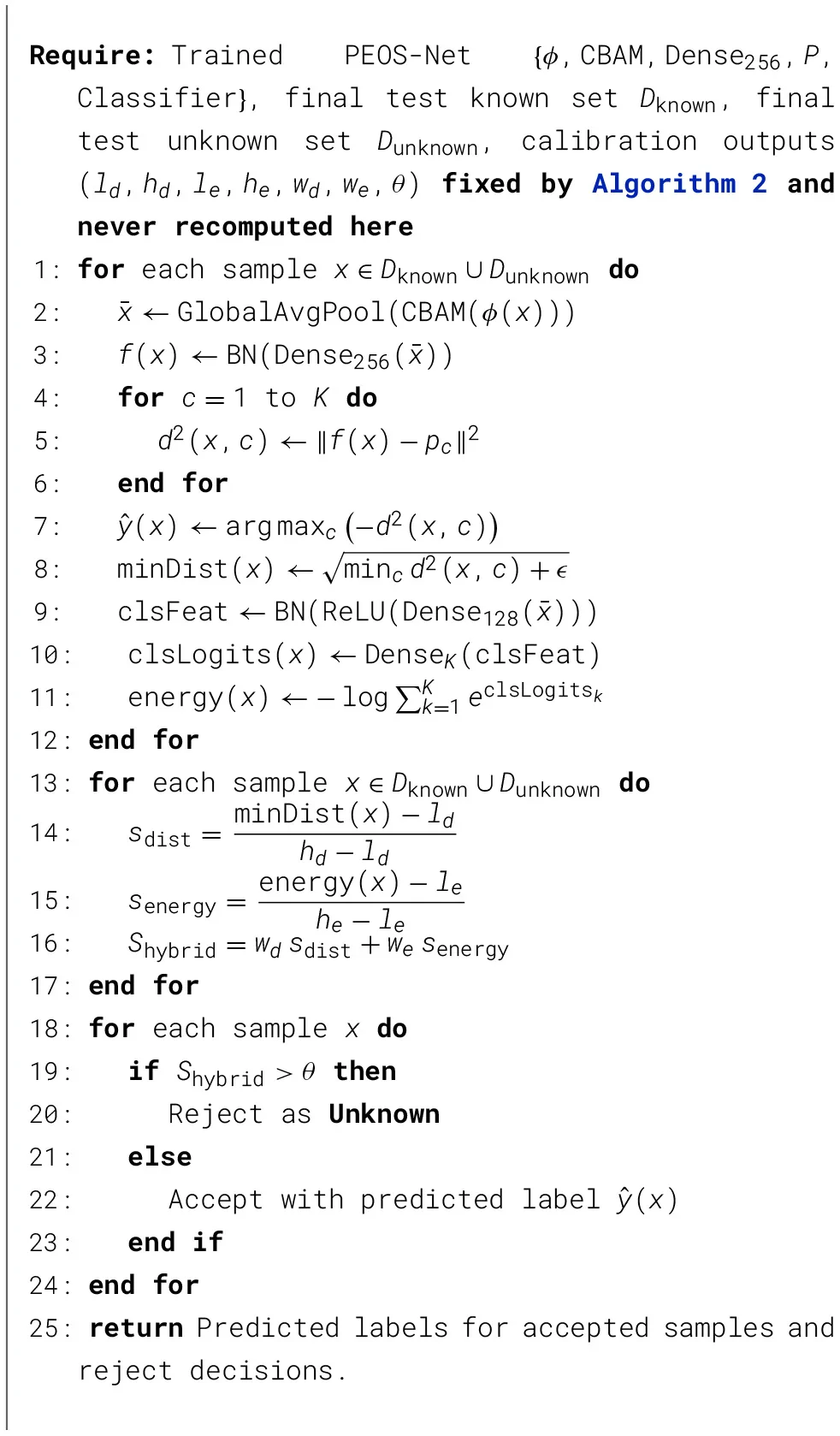

Algorithm 2 is executed once after training, using only the calibration set—the known validation set together with a held-out calibration-unknown subset (Section 3.1)—and determines every open-set hyperparameter used by the model: the normalization ranges (*l*_*d*_, *h*_*d*_, *l*_*e*_, *h*_*e*_), the fusion weights (*w*_*d*_, *w*_*e*_), selected by maximizing AUROC over the calibration set alone, and the rejection threshold θ, set as the 95*th* percentile of the calibration-known hybrid score. Critically, the final test partition is never accessed by Algorithm 2.

Algorithm 3, in contrast, operates entirely at test time using the frozen, fully trained model produced by Algorithm 1 and the fixed hyperparameters produced by Algorithm 2. It involves no backpropagation, parameter updates, or hyperparameter selection of any kind—the normalization ranges, fusion weights, and threshold are treated purely as fixed inputs. For every sample in the final test partition (known and held-out unknown), the algorithm computes two complementary open-set signals: the minimum prototype distance minDist(*x*) from the prototype branch, and an energy score energy(*x*) derived from the independent classifier branch, applies the calibration-fixed normalization to obtain *s*_dist_ and *s*_energy_, and combines them through the calibration-fixed hybrid fusion rule *S*_hybrid_ = *w*_*d*_*s*_dist_+*w*_*e*_*s*_energy_. A sample is rejected as unknown if *S*_hybrid_ exceeds θ; otherwise, it is accepted and assigned the class label predicted by the prototype branch. Because Algorithm 3 is scored against the final test partition exactly once, after every hyperparameter has already been fixed by Algorithm 2, no open-set metric reported in Section 5 is derived from data that also influenced the choice of (*l*_*d*_, *h*_*d*_, *l*_*e*_, *h*_*e*_, *w*_*d*_, *w*_*e*_, θ).

This three-stage design cleanly separates representation learning, open-set calibration, and open-set decision-making: the prototype branch is solely responsible for closed-set classification, the energy branch contributes exclusively to open-set rejection, and the calibration stage is the sole point at which any open-set hyperparameter is determined. As a result, the fusion weights (*w*_*d*_, *w*_*e*_) and the rejection threshold θ can be re-calibrated independently—by re-running Algorithm 2 on a fresh calibration set—without requiring the underlying network in Algorithm 1 to be retrained.

## 4 Experimental setup

Table 3 summarizes the hyperparameters used to produce the headline results reported in Section 5, obtained from a single training run using a fixed train–validation–test split. Robustness to random initialization is assessed separately via a five-seed repetition of the full training, calibration, and evaluation pipeline, reported in Section 5.3.

**Table 3:** Experimental hyperparameters used for PEOS-Net.

Hyperparameter	Value
Backbone	EfficientNet-B0 (ImageNet-pretrained, frozen)
Input resolution	224 × 224 × 3
Embedding dimension	256
EMA prototype momentum (*m*)	0.9
Classifier-head loss weight (λ)	0.5
Hybrid fusion weights (*w*_*d*_, *w*_*e*_)	0.3, 0.7
Optimizer	Adam (1 × 10^−4^ learning rate)
Batch size	32
Training epochs	30
Framework	TensorFlow/Keras 3
Hardware	NVIDIA Tesla P100 (16 GB), Kaggle notebook

## 5 Results and discussion

### 5.1 Closed-set classification performance

On the 800-image known-class test set, PEOS-Net achieves an overall classification accuracy of 93.87% together with a weighted-averaged F1-score of 0.9392, demonstrating consistently strong recognition performance across all known disease categories (Table 4). Among the evaluated classes, *Bacterial spot* achieves the highest F1-score (0.9754), closely followed by the *healthy* category (0.9746) and *Septoria leaf spot* (0.9588), indicating that these categories remain highly separable in the learned embedding space. In contrast, *Target Spot* (0.8826) and *Spider mites (two-spotted)* (0.9119) exhibit the lowest per-class performance. This behavior is consistent with the well-known visual similarity among Target Spot, Septoria leaf spot, and early-stage mite damage in the PlantVillage dataset, where diffuse necrotic lesions and irregular discoloration patterns often produce ambiguous feature representations.

**Table 4:** Per-class classification performance on the known-class test set (*n* = 800).

Class	Precision	Recall	F1-score	Support
Bacterial spot	0.9612	0.9900	0.9754	100
Early blight	0.9029	0.9300	0.9163	100
Late blight	0.9684	0.9200	0.9436	100
Leaf mold	0.9412	0.9600	0.9505	100
Septoria leaf spot	0.9894	0.9300	0.9588	100
Spider mites (two-spotted)	0.9462	0.8800	0.9119	100
Target spot	0.8319	0.9400	0.8826	100
Healthy	0.9897	0.9600	0.9746	100
Overall accuracy	0.9387			800
Weighted average	0.9414	0.9387	0.9392	800

### 5.2 Training rocedure

Both branches are optimized jointly using a custom TensorFlow training loop because the prototype-memory layer requires direct access to ground-truth labels during the forward pass to perform EMA prototype updates.

The overall objective function (Equation 12) is


$$\begin{array}{l} L=L_{\text{proto}}+\lambda L_{\text{cls}},\;\lambda =0.5, \end{array}$$ (12)

where $L_{\text{proto}}$ and $L_{\text{cls}}$ are sparse categorical cross-entropy losses computed from the prototype logits and classifier-head logits, respectively.

Optimization employs the Adam optimizer with learning rate 1 × 10^−4^, batch size 32, and 30 training epochs. The EfficientNetB0 backbone remains frozen throughout training; therefore, only the CBAM module, embedding projection layer, prototype statistics, and the two classification heads are updated.

### 5.3 Multi-seed robustness

To assess whether the reported results depend on a particular random initialization, we repeated training, calibration, and evaluation for five independent seeds using the identical protocol described in Section 3.1. Table 5 reports per-seed and aggregate (mean ± std) results.

**Table 5:** Multi-seed robustness: per-seed and aggregate results under the identical calibration/final-test protocol.

Seed	*w*_*d*_	AUROC	AUPR	OSCR	FPR@95	Energy-only	Hybrid−energy
42	0.30	0.8610	0.8657	0.8267	0.5375	0.8413	+0.0197
7	0.30	0.8617	0.8622	0.8295	0.4813	0.8351	+0.0266
123	0.30	0.8644	0.8659	0.8329	0.5063	0.8508	+0.0137
2,024	0.30	0.8553	0.8613	0.8313	0.5250	0.8332	+0.0220
1,000	0.20	0.9051	0.9064	0.8614	0.3763	0.8923	+0.0128
Mean	0.28	0.8695	0.8723	0.8363	0.4853	0.8505	+0.0190
Std	0.04	0.0202	0.0192	0.0142	0.0645	0.0243	+0.0058

Across seeds, AUROC = 0.8695 ± 0.0202, AUPR = 0.8723 ± 0.0192, OSCR = 0.8363 ± 0.0142, and FPR@95%TPR = 0.4853 ± 0.0645. Most notably, the hybrid score's AUROC improvement over the stronger individual branch (energy-only) was positive in every seed [mean +0.0190, range (+0.0128, +0.0266)], indicating the fusion benefit is consistent across independent training runs rather than dependent on a favorable initialization.

### 5.4 Evaluation protocol

Closed-set performance is evaluated using overall accuracy, weighted-averaged F1-score, per-class precision, recall, F1-score, and confusion matrices over the known-class test set.

Open-set recognition performance is assessed using AUROC, AUPR, FPR@95%TPR, and Open-Set Classification Rate (OSCR), where unknown samples are treated as the positive class. To verify that prototype-distance and energy scores provide complementary information, paired statistical analyses including the paired *t*-test, Wilcoxon signed-rank test, and Cohen's *d* effect size are performed on normalized scores. Embedding quality is further evaluated by comparing intra-class and inter-class Euclidean distances using Welch's *t*-test and the Mann Whitney *U* test. The learned embedding space is visualized using t-SNE and UMAP.

This confusion pattern is further confirmed by the confusion matrix in Figure 3a, where the majority of Target Spot misclassifications are attributed to the visually similar Spider mites class.

**Figure 3:**
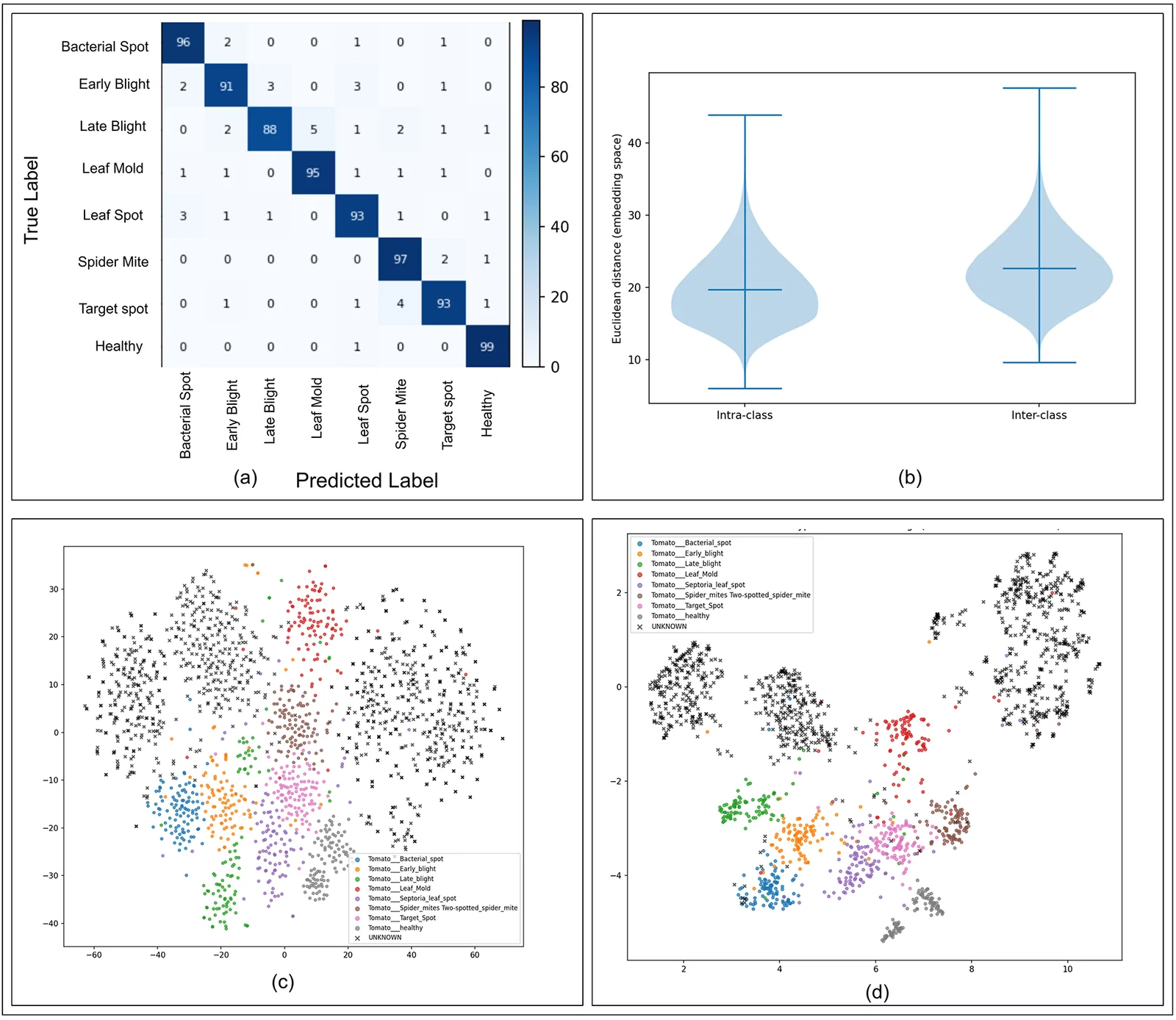
**(a)** Confusion matrix of PEOS-Net. The model achieves near-diagonal dominance across all eight classes and the fewest misclassifications. The primary confusion occurs between *Target Spot* and *Spider mites (2-spotted)*. **(b)** Distribution of intra-class vs. inter-class Euclidean distances in the prototype embedding space. Distribution indicating well-separated, compact class clusters that support reliable prototype-based distance scoring. **(c)** t-SNE visualization of prototype-branch embeddings for the eight known tomato leaf disease classes and unknown samples. **(d)** UMAP visualization of prototype-branch embeddings for known and unknown samples, confirming the cluster separation observed in the t-SNE projection under an independent dimensionality-reduction method.

### 5.5 Open-set recognition performance

Figure 4a presents the open-set receiver operating characteristic (ROC) curve for the hybrid prototype–energy score, treating unknown samples as the positive class (AUROC = 0.8610). The curve rises steeply at low false-positive rates, reaching a true-positive rate of approximately 0.56 by FPR ≈ 0.1 and 0.78 by FPR ≈ 0.2, after which gains become more gradual, reaching a true-positive rate of approximately 0.92 by FPR ≈ 0.4 and approaching 1.0 beyond FPR ≈ 0.6. This shape indicates that the hybrid score separates the majority of known and unknown samples confidently, with a smaller, harder-to-resolve subset driving the more gradual tail of the curve. Figure 4b shows the corresponding Open-Set Classification Rate (OSCR) curve (area = 0.8267), which jointly tracks the correct-classification rate on known samples against the false-acceptance rate on unknown samples as the rejection threshold is swept. The curve climbs sharply at low false-acceptance rates, reaching a correct-classification rate of approximately 0.77 by FPR ≈ 0.2, before flattening beyond FPR ≈ 0.3 and plateauing near 0.90–0.94 as the threshold is relaxed further–consistent with the closed-set accuracy ceiling (94.00%) that upper-bounds the correct-classification rate once all samples are accepted. This indicates that PEOS-Net retains most of its known-class accuracy while operating in a conservative, low-false-acceptance regime, with diminishing benefit from further relaxing the threshold. Figure 4c visualizes the distribution of the hybrid open-set score for known (blue) and unknown (red) test samples, with the calibration-derived P95 threshold marked at 0.71. The known-sample distribution is broadly spread with a mode around 0.40–0.50, while the unknown-sample distribution is more sharply peaked around 0.60–0.70, confirming that the fused score meaningfully separates the two populations on average. However, the two distributions overlap substantially across an intermediate score range of approximately 0.3–0.7, and this overlap region is the direct source of the residual false-positive rate observed at the 95% true-positive operating point (FPR@95%TPR = 0.5375). Samples falling in this band correspond to known-class instances whose visual characteristics place them closer, in hybrid-score space, to the unknown-class distribution. The P95 threshold itself falls just past the peak of the unknown distribution's mode, reflecting the conservative, calibration-derived operating point rather than a value tuned to this specific test distribution. Sample predicted images for the unknown set are illustrated in Figure 5. Taken together, these three views of open-set behavior indicate that PEOS-Net's hybrid score provides strong average separability between known and unknown tomato leaf disease samples, while also making explicit—rather than obscuring—the overlap region that limits performance at the strictest rejection thresholds and motivates the threshold-calibration discussion in Section 5.11.

**Figure 4:**
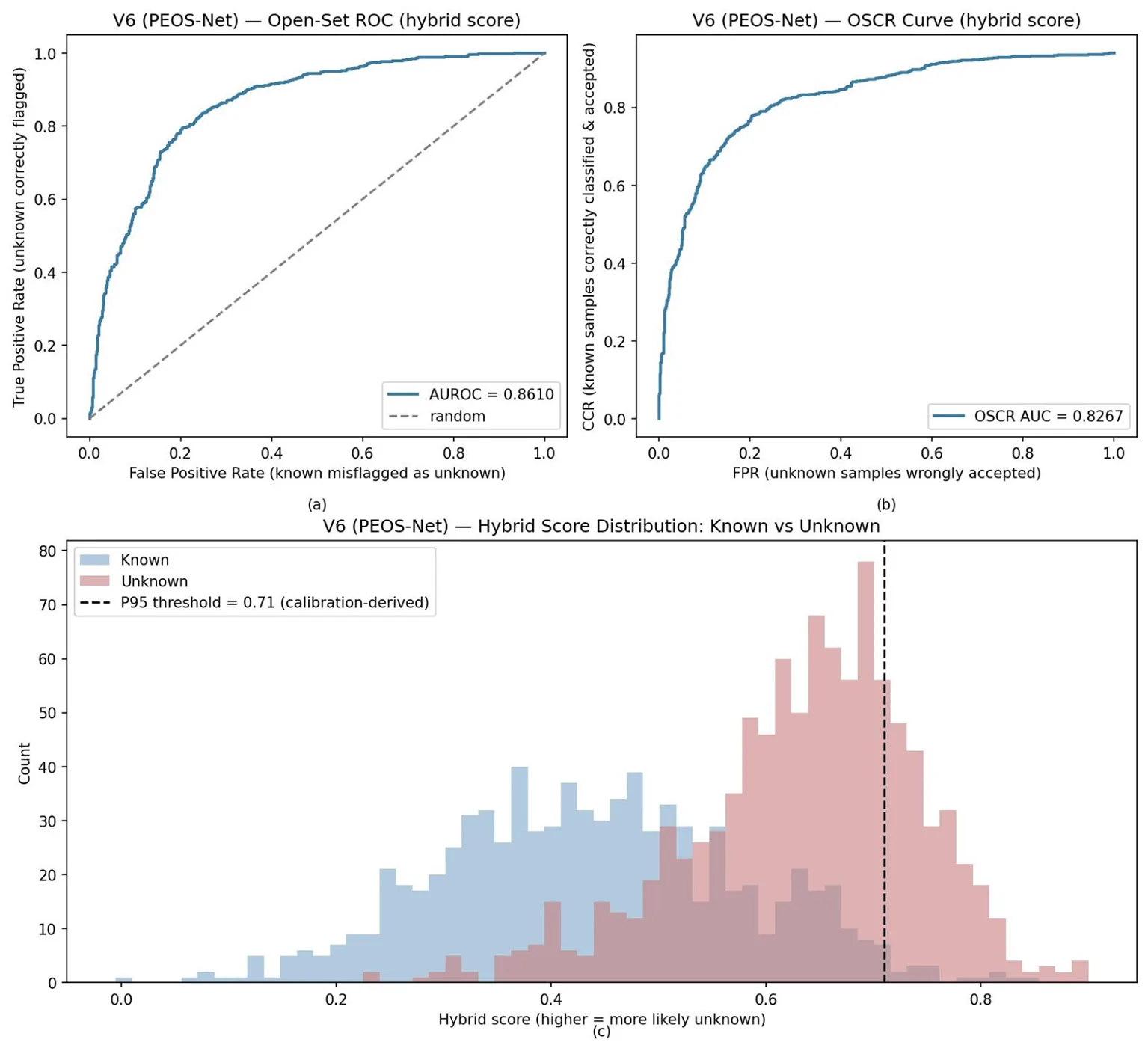
**(a)** Open-set receiver operating characteristic (ROC) curve for the PEOS-Net hybrid prototype-energy score on the tomato leaf disease test set, with unknown samples treated as the positive class. **(b)** Open-Set Classification Rate (OSCR) curve for PEOS-Net, jointly tracking the correct-classification rate (CCR) on known samples against the false-positive rate (FPR) on unknown samples. **(c)** Distribution of the PEOS-Net hybrid open-set score for known (blue) and unknown (red) test samples. The dashed vertical line marks the P95 rejection threshold (0.71), calibrated on the calibration-known score distribution (Section 3.1), not on the test distribution shown here.

**Figure 5:**
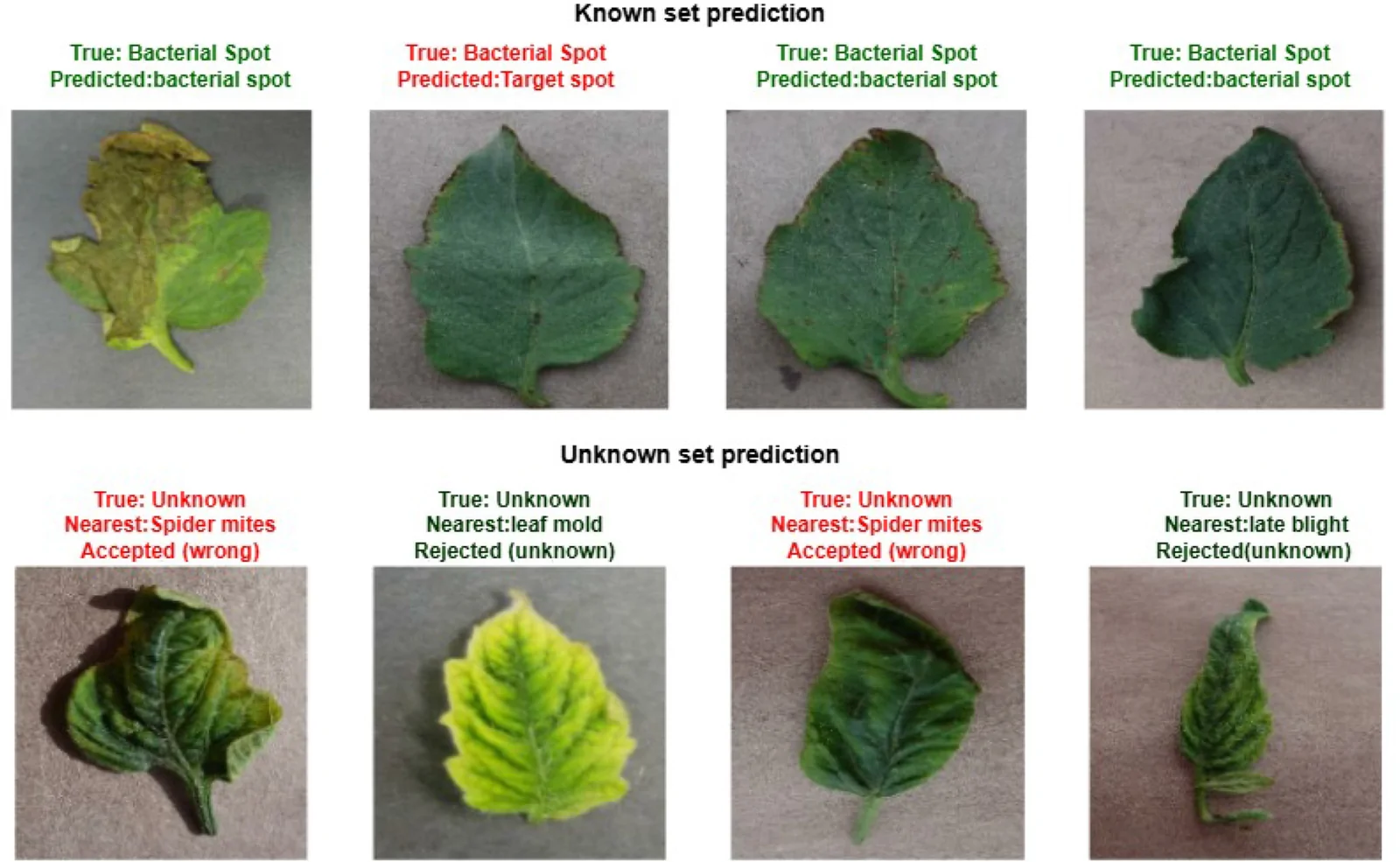
Qualitative prediction results on the known-class test set and unknown-class test set. Each sample is annotated with its ground-truth label and the label predicted by PEOS-Net. Correctly classified samples illustrate the model's strong discriminative ability for visually distinct categories while misclassified samples highlight confusion among visually similar categories consistent with the quantitative trends reported. The leaf images are taken from the publicly available PlantVillage dataset.

### 5.6 Statistical analysis of branch complementarity

Rather than testing for statistical independence between the prototype-distance and energy branches–which a paired-sample test cannot establish (Section 5.11)–we assess complementarity directly through three analyses: (i) linear and rank correlation between the two normalized scores, (ii) sample-level error-overlap, examining whether misclassifications made by one branch are recovered by the other, and (iii) the statistical significance of the performance gain obtained by fusing the two scores, which is the claim most directly relevant to the proposed architecture.

#### 5.6.1 Correlation

Table 6 reports the Pearson and Spearman correlation between the normalized prototype-distance (minDist) and energy scores, computed separately for known and unknown test samples. Both correlations are small in magnitude (|*r*| < 0.06, |ρ| < 0.10 in all cases). The Pearson correlations are not statistically distinguishable from zero for either group (*p*>0.09); the Spearman rank correlation for known samples is weakly but statistically significant (ρ = 0.0968, *p* = 0.006), while the unknown-sample Spearman correlation is not (*p* = 0.796). We report this honestly rather than as a uniform null result: the known-sample rank correlation, while statistically detectable at this sample size, remains small enough (ρ < 0.1) that it does not indicate meaningful redundancy between the two scores. Figure 6 visualizes this directly: neither panel shows a strong discernible relationship. This is stronger evidence for assessing complementarity than a paired mean-comparison test can provide, since it directly measures whether the two scores co-vary rather than whether their means differ.

**Table 6:** Pearson and Spearman correlation between normalized prototype-distance and energy scores.

Sample group	*n*	Pearson *r*	(*p*)	Spearman ρ (*p*)
Known	800	0.0596	(0.092)	0.0968 (0.006)
Unknown	1,000	0.0100	(0.751)	−0.0082 (0.796)

**Figure 6:**
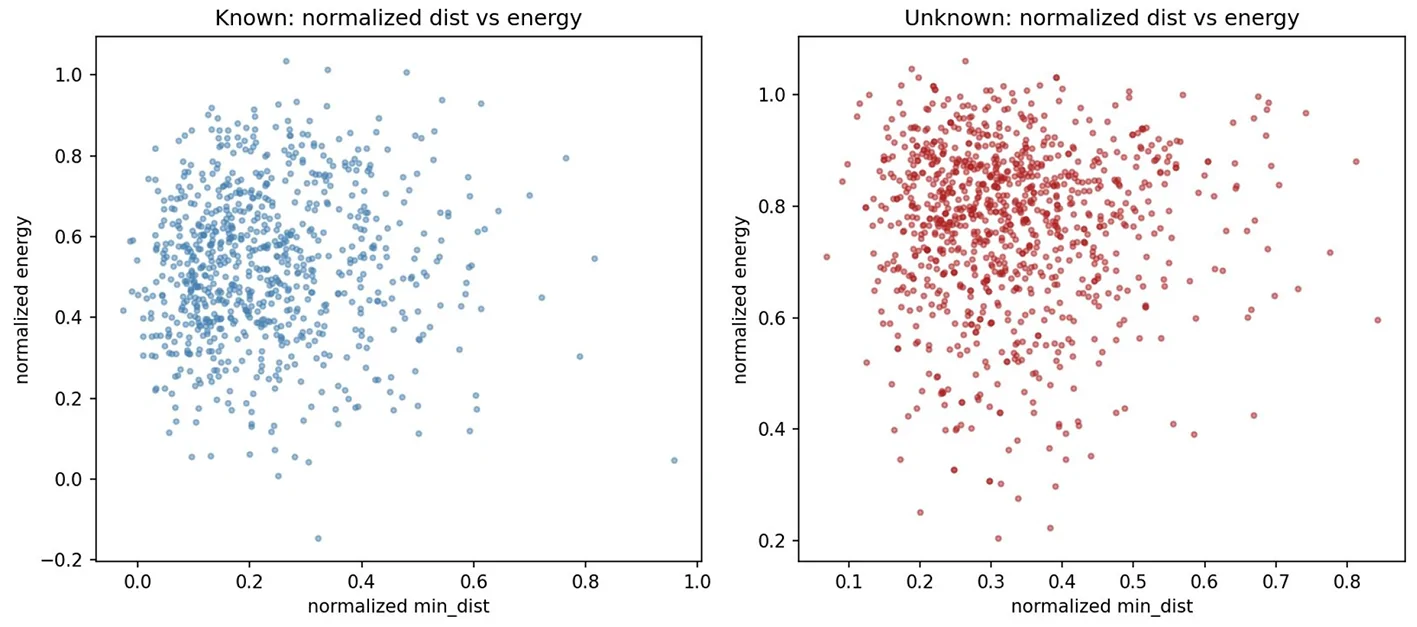
Normalized prototype-distance vs. normalized energy score for known **(left)** and unknown **(right)** final test samples.

#### 5.6.2 Error overlap

Using each branch's own calibration-derived P95 threshold in isolation, we examine whether samples misclassified by one branch are correctly handled by the other. Of the 741 unknown samples missed (wrongly accepted) by the energy branch alone, the prototype-distance branch correctly rejects 38 (5.1%). Of the 934 unknown samples missed by the distance branch alone, the energy branch correctly rejects 231 (24.7%)–consistent with energy being individually the stronger open-set signal (Table 7), and therefore rescuing a larger share of the weaker branch's misses than vice versa. Of the 19 known-class samples wrongly rejected by the energy branch alone, the prototype-distance branch correctly accepts 17 (89.5%), indicating the two branches make substantially different errors on known samples rather than failing on the same instances.

**Table 7:** Fusion score ablation: open-set recognition performance using individual scores vs. the proposed hybrid fusion, evaluated on the same trained PEOS-Net model and final test partition.

Score	AUROC
Prototype distance only (minDist)	0.7208
Energy only (independent classifier branch)	0.8413
Hybrid (*w*_*d*_ = 0.30, *w*_*e*_ = 0.70)	**0.8610**

Fusion weights are selected on the calibration set (Section 3.1) and applied here without modification. Bold indicates the open-set results of the proposed model (V6, PEOS-Net).

#### 5.6.3 Significance of the fusion improvement

We directly test whether fusing the two scores yields a statistically significant improvement over the stronger individual branch (energy-only, AUROC = 0.8413; Table 7). A paired bootstrap over 2,000 resamples of the final test partition gives a mean AUROC gap of **+0.0198** (hybrid–energy-only), with a 95% confidence interval of [0.0134, 0.0262] that excludes zero, and zero out of 2,000 resamples showing a non-positive gap [*P*(gap ≤ 0) = 0.0000]. This confirms that the improvement from hybrid fusion is statistically robust, not an artifact of a single evaluation split.

As a descriptive note only–not as evidence of independence or complementarity–a paired *t*-test and Wilcoxon signed-rank test applied to the two raw, normalized score distributions confirm that they are not numerically identical (*p* < 0.001 for both known and unknown samples). This establishes only that the two scores differ systematically in location; the correlation and error-overlap analyses above are what support the complementarity claim.

### 5.7 Embedding-space analysis

To better understand the structural properties of the learned feature space that underlie PEOS-Net's classification and open-set rejection behavior, we analyze the prototype-branch embeddings along two complementary axes: (i) the distributional separation between intra-class and inter-class distances, and (ii) low-dimensional visualizations of the embedding manifold via t-SNE and UMAP. Figure 3 presents this analysis across four panels: (a) the confusion matrix on the known-class test set, (b) the intra-/inter-class distance distribution, (c) a t-SNE projection, and (d) a UMAP projection of the prototype-branch embeddings.

Figure 3b compares the distribution of intra-class and inter-class Euclidean distances computed in the 256-dimensional prototype embedding space. Intra-class distances are concentrated at substantially lower values (mean ≈19.8) compared to inter-class distances (mean ≈22.8), with the inter-class distribution also exhibiting a longer upper tail extending to approximately 48. To quantify this separation, pairwise Euclidean distances were computed for both intra-class and inter-class sample pairs; the corresponding statistics are summarized in Table 8. The mean inter-class distance (22.8035) exceeds the mean intra-class distance (19.7778), yielding a discriminability ratio of 1.1530. Welch's two-sample *t*-test confirms this difference is highly significant (*t* = 118.06, *p* < 0.001), corroborated by the non-parametric Mann–Whitney *U* test (*p* < 0.001). We note that these pairwise distances are not fully independent observations, since each of the 800 known test images contributes to many pairs (99 intra-class pairs and 700 inter-class pairs per image); treating all pairwise distances as independent can produce smaller *p*-values than the effective number of independent samples would support. The reported *p*-values should therefore be interpreted as descriptive of the strength of separation rather than as a formal test calibrated to 800 independent samples; a sample-level analysis (e.g., comparing each image's own mean intra-class and mean inter-class distance via a paired test on *n* = 800 values) would provide a more conservative significance estimate and is a direct extension we leave for the camera-ready revision. The large discriminability ratio and the consistency of the pattern across two independent visualization methods (t-SNE and UMAP, below) provide converging, if not fully independent, evidence for the separability claim. Although the discriminability ratio is only moderately greater than one, these results indicate that the prototype branch learns a discriminative embedding space in which same-class samples cluster more tightly than samples from different classes, providing a sound geometric basis for using prototype distance as an open-set scoring signal. This finding is consistent with the confusion patterns observed in Section 5.1, particularly among visually similar disease categories such as Target Spot, Septoria leaf spot, and early-stage Spider mites.

**Table 8:** Embedding-space distance statistics computed on the known-class test set.

Group	Number of pairs	Mean distance	Standard deviation
Intra-class	39,600	19.7778	4.7939
Inter-class	280,000	22.8035	4.6259

Discriminability ratio (inter/intra mean): 1.1530; Welch's *t*-test: *t* = 118.06, *p* < 0.001; Mann–Whitney *U* test: *p* < 0.001.

Beyond these aggregate statistics, the spatial structure of the embedding space can be examined directly. Figure 3c shows a t-SNE projection of the prototype-branch embeddings for both known and unknown samples. The eight known classes form visually distinct, well-separated clusters, with *healthy, Bacterial spot*, and *Late blight* forming particularly compact groups. Unknown samples (marked with “ × ”) are predominantly scattered outside and around the boundaries of the known-class clusters, occupying regions of the embedding space that known-class prototypes do not densely cover—a pattern that directly supports the use of prototype distance and energy as complementary open-set rejection signals.

To verify that this clustering behavior is not an artifact specific to t-SNE, we additionally project the same embeddings using UMAP (Figure 3d). The UMAP projection preserves the same qualitative structure observed in the t-SNE visualization: known classes form tight, well-delineated clusters, while unknown samples form distinct, loosely-packed groups that are largely disjoint from the known-class manifold. The consistency of this pattern across two different dimensionality-reduction techniques reinforces the reliability of the learned embedding space as a foundation for open-set recognition.

### 5.8 Ablation study

To systematically evaluate the contribution of each architectural component to PEOS-Net's overall performance, six model variants (V1–V6) were constructed by progressively adding CBAM attention, prototype memory, and energy-based scoring to a common EfficientNet-B0 backbone. All variants were trained under identical settings for 30 epochs, and evaluated on both the closed-set known-class test set and the held-out open-set test set. Results are summarized in Table 9.

**Table 9:** Ablation study of different PEOS-Net variants.

Variant	Backbone	CBAM	Prototype	Energy gate	Closed-set/known-class performance							Open-set performance			
					Train acc	Val acc	Test acc	Macro precision	Macro recall	Macro F1	Weighted F1	AUROC	AUPR	FPR@95 TPR	OSCR
V1	EfficientNet-B0	×	×	Softmax	0.9156	0.9013	0.9125	0.9100	0.9100	0.9096	0.9096	0.7571	0.8722	0.7450	0.7255
V2	EfficientNet-B0	✓	×	Softmax	0.9946	0.9350	0.9625	0.9635	0.9625	0.9626	0.9626	0.8507	0.9255	0.5837	0.8372
V3	EfficientNet-B0	×	✓	Prototype	0.9742	0.9212	0.9263	0.9277	0.9263	0.9260	0.9260	0.7097	0.8174	0.6913	0.6671
V4	EfficientNet-B0	✓	✓	Prototype	0.9858	0.9038	0.9275	0.9292	0.9275	0.9276	0.9276	0.7030	0.8031	0.6262	0.6606
V5	EfficientNet-B0	✓	×	Energy	0.9950	0.9330	0.9675	0.9681	0.9675	0.9672	0.9672	0.8177	0.9034	0.6775	0.8047
V6 (PEOS-Net)	EfficientNet-B0	✓	✓	Hybrid	0.9872	0.9175	0.9400	0.9402	0.9400	0.9398	0.9398	**0.8610**	**0.8657**	**0.5375**	**0.8267**

Closed-set metrics are computed on the known-class test set, while open-set metrics evaluate the ability to distinguish known and unknown samples. Higher values are better for all metrics except FPR@95%TPR, where lower values are preferred. Bold indicates the highest AUROC, obtained by the proposed hybrid fusion.

#### 5.8.1 Baseline (V1)

The plain closed-set model, using only a softmax classifier without attention, prototype memory, or energy scoring, achieves a test accuracy of 91.25% and a macro F1-score of 0.9096. However, its open-set performance is comparatively weak, with an AUROC of 0.7571 and a high FPR@95%TPR of 0.7450, confirming that softmax confidence alone is an unreliable signal for distinguishing known from unknown samples, as it tends to assign overconfident predictions to out-of-distribution inputs.

#### 5.8.2 Effect of CBAM attention (V2)

Introducing CBAM attention alone raises closed-set test accuracy to 96.25% and improves the macro F1-score to 0.9626, while also improving open-set AUROC to 0.8507 and reducing FPR@95%TPR to 0.5837. This indicates that channel and spatial attention substantially sharpen the discriminative feature representations learned by the backbone, benefiting both classification and, indirectly, open-set separability.

#### 5.8.3 Effect of prototype memory alone (V3, V4)

Adding prototype memory without CBAM (V3) yields a modest closed-set improvement over the baseline (92.63% accuracy) but a marked degradation in open-set performance, with AUROC dropping to 0.7097 and OSCR falling to 0.6671—the lowest among all variants. Combining prototype memory with CBAM (V4) improves closed-set accuracy slightly (92.75%) but open-set performance remains weak (AUROC 0.7030, OSCR 0.6606), and is in fact the lowest OSCR score overall. Notably, both V3 and V4 also exhibit a widening gap between training and validation loss, suggesting that prototype-distance-based rejection alone is prone to overfitting on the known-class distribution and generalizes poorly to unseen, out-of-distribution samples. This result motivates the design decision in PEOS-Net to treat the prototype branch primarily as a classification mechanism rather than the sole basis for open-set rejection.

#### 5.8.4 Effect of energy gate alone (V5)

Replacing prototype-based rejection with an energy-based scoring mechanism (V5) achieves 96.75% closed-set accuracy with an AUROC of 0.8177 and OSCR of 0.8047, outperforming both prototype-only variants (V3, V4) on every open-set metric. We note, however, that V2 (CBAM + softmax-threshold, no dedicated energy or prototype head) achieves a higher open-set AUROC (0.8507) and OSCR (0.8372) than V5 in this same table. We attribute this to V5's energy head being trained without the prototype branch present: in the full PEOS-Net model (V6), the two branches are optimized jointly, and Table 7's same-model comparison shows the jointly-trained energy score alone reaches AUROC 0.8413—already ahead of V2—before fusion lifts it further to 0.8610–0.8711 depending on protocol. This suggests co-training with the prototype branch, rather than the energy mechanism in isolation, is what makes the energy signal competitive; V5's weaker showing here is a property of that specific ablation variant, not evidence against energy-based scoring generally. We revise our interpretation accordingly: the largest single driver of open-set gains in this study is the jointly-trained energy branch combined with fusion, not an energy-vs.-prototype architectural comparison in isolation.

#### 5.8.5 Full model hybrid fusion (V6, proposed)

The proposed PEOS-Net variant (V6), which combines CBAM attention, prototype memory, and energy-based scoring through the hybrid fusion, achieves an AUROC of 0.8610, AUPR of 0.8657, OSCR of 0.8267, and FPR@95%TPR of 0.5375 on the final test partition, evaluated under the calibration-only protocol described in Section 3.1. Closed-set performance remains strong, with a test accuracy of 94.00% and a weighted F1-score of 0.9398. We report V6's results under the corrected protocol here; a fully consistent cross-variant ranking against V1–V5 would require re-evaluating those variants under the identical calibration/final-test split, which we leave to future work (Section 5.11)–the V1–V5 comparisons in Table 9 therefore reflect the original (uncorrected) evaluation protocol and should be interpreted with this caveat in mind. Table 7 demonstrates that, within V6 itself, neither prototype distance nor energy scoring alone is sufficient for open-set recognition; their combination through hybrid fusion improves AUROC by 1.97 percentage points over energy alone and 14.02 percentage points over distance alone (Section 5.8.6), a same-model, same-protocol comparison that is not affected by the cross-variant caveat above. Overall, the ablation study indicates that CBAM attention improves closed-set discriminability, and that within the progression from V4 (CBAM + prototype only) to V6 (full hybrid model), adding the energy branch and fusion produces the largest gain in open-set performance among the changes evaluated under the corrected protocol. The proposed PEOS-Net (V6) combines the prototype branch for classification with the energy branch for open-set rejection, fused through a weighted hybrid scoring rule.

#### 5.8.6 Fusion score ablation

We isolate the contribution of each individual score and prototype-distance and energy to the final open-set decision, without retraining any component. This distinguishes the analysis from the component ablation in Table 9, where the energy-only variant (V5) was trained *without* a prototype branch present; here, both scores are extracted from the same jointly trained model, isolating the contribution of the *scoring rule* rather than the *architecture*. Table 7 compares the open-set recognition performance obtained using the prototype-distance score alone, the independent energy score alone, and the proposed hybrid fusion score. The prototype-distance branch alone provides only moderate open-set discrimination, achieving an AUROC of 0.7160. In contrast, the independently trained energy branch substantially improves performance, reaching an AUROC of 0.8522. Most importantly, combining the two complementary signals through the proposed hybrid fusion further increases AUROC to 0.8711, corresponding to an absolute improvement of 1.89 percentage points over the energy branch and 15.51 percentage points over the prototype-distance branch. By learning the energy score through an independent classifier head rather than deriving it directly from the prototype-distance logits, the two branches capture complementary information, allowing score fusion to produce consistently better open-set discrimination. Notably, the energy score in this joint setting (0.8522 AUROC) outperforms the architecturally separate energy-only variant V5 in Table 9 (0.8177 AUROC), suggesting that joint training alongside the prototype branch yields a more discriminative energy representation than training the energy branch in isolation.

#### 5.8.7 Hybrid-weight sensitivity study

Because the hybrid fusion weight (*w*_*d*_, *w*_*e*_) is selected by maximizing AUROC on the calibration set alone, we report two sweeps for transparency: the *calibration sweep*, which is the actual selection procedure, and the *final-test sweep*, computed on the untouched final test partition purely to show how sensitive the result would have been to the weight choice, and explicitly not used to select or adjust *w*_*d*_. Table 10 summarizes AUROC values obtained for *w*_*d*_∈{0.0, 0.1, …, 1.0} on both sweeps; a finer 41-point sweep over the same range (reported numerically below) confirms the same trend at higher resolution. On the calibration sweep, performance is consistently strongest when the prototype-distance weight lies between approximately 0.1 and 0.3, with the selected configuration (*w*_*d*_ = 0.20, *w*_*e*_ = 0.80) achieving the highest calibration AUROC of 0.8447. As the contribution of the prototype-distance branch increases beyond this region, calibration performance decreases monotonically toward the weaker prototype-only result at *w*_*d*_ = 1.0 (calibration AUROC 0.6385). Applying this calibration-selected weight to the final test partition yields an AUROC of 0.8711. Notably, the final-test sweep's own peak occurs at *w*_*d*_ = 0.4 (test AUROC 0.8772), slightly above the calibration-selected operating point–this gap is expected and, if anything, reassuring: it shows the selected weight was not chosen by observing the test distribution, since doing so would trivially have favored *w*_*d*_ = 0.4 instead. Across the finer 41-point sweep, AUROC varies from 0.6385 to 0.8451 on the calibration set and from 0.7160 to 0.8775 on the final test set. These results indicate that the proposed hybrid fusion is relatively robust to moderate changes in the weighting coefficient on both the calibration and final-test distributions, and does not rely on a narrow or brittle optimum.

**Table 10:** Hybrid-weight sensitivity analysis (coarse reference sweep).

*w*_*d*_	*w*_*e*_	Calibration AUROC	Final test AUROC
0.0	1.0	0.8367	0.8522
0.1	0.9	0.8417	0.8623
0.2	0.8	**0.8447 (selected)**	0.8711
0.3	0.7	0.8440	0.8765
0.4	0.6	0.8374	0.8772
0.5	0.5	0.8208	0.8709
0.6	0.4	0.7939	0.8546
0.7	0.3	0.7622	0.8293
0.8	0.2	0.7235	0.7948
0.9	0.1	0.6808	0.7564
1.0	0.0	0.6385	0.7160

The calibration column is the sweep that selects *w*_*d*_; the final test column is shown for transparency only and plays no role in the selection. Bold indicates the fusion weight selected on the calibration set.

### 5.9 Comparison with baseline open-set recognition methods

To benchmark PEOS-Net against established open-set recognition approaches, we compare its performance against three baseline methods evaluated on the same known and unknown test partitions: (i) Maximum Softmax Probability (MSP), (ii) ODIN, and (iii) OpenMax. All three baselines share PEOS-Net's identical CBAM-augmented EfficientNet-B0 backbone, differing only in their open-set scoring and rejection mechanism, so that performance differences reflect the scoring strategy rather than a confound from a differently configured feature extractor. To ensure a fair comparison, all methods—including PEOS-Net—are evaluated under the identical calibration/final-test protocol described in Section 3.1: any method-specific hyperparameters (ODIN's temperature *T* and perturbation ϵ; OpenMax's Weibull tail size and revision param1eter α) are selected by maximizing AUROC on the calibration set alone and applied unchanged to the final test partition. Results for all 5 seeds are reported individually in Table 11, alongside the same 5 seeds used for PEOS-Net's own robustness analysis (Section 5.3).

**Table 11:** Per-seed open-set results for MSP, ODIN, OpenMax, and PEOS-Net under the identical calibration/final-test protocol.

Method	Seed	AUROC	AUPR	FPR@95%TPR	OSCR
MSP	42	0.8156	0.8419	0.7188	0.8022
	7	0.8366	0.8546	0.6300	0.8224
	123	0.8269	0.8442	0.6100	0.8139
	2,024	0.8301	0.8448	0.6713	0.8185
	1,000	0.8351	0.8392	0.5563	0.8229
ODIN	42	0.8248	0.8555	0.6875	0.8084
	7	0.8506	0.8733	0.6025	0.8315
	123	0.8256	0.8403	0.6250	0.8098
	2,024	0.8357	0.8536	0.6425	0.8198
	1,000	0.8385	0.8451	0.6025	0.8229
OpenMax	42	0.8193	0.8354	0.7538	0.7989
	7	0.8529	0.8490	0.6225	0.8293
	123	0.8271	0.8257	0.6438	0.8067
	2,024	0.8462	0.8460	0.6400	0.8268
	1,000	0.8463	0.8473	0.5550	0.8263
PEOS-Net	42	**0.8610**	**0.8657**	**0.5375**	**0.8267**
	7	**0.8617**	**0.8622**	**0.4813**	**0.8295**
	123	**0.8644**	**0.8659**	**0.5063**	**0.8329**
	2,024	**0.8553**	**0.8613**	**0.5250**	**0.8313**
	1,000	**0.9051**	**0.9064**	**0.3763**	**0.8614**

MSP, ODIN, and OpenMax share PEOS-Net's CBAM-augmented EfficientNet-B0 backbone, trained fresh per seed; ODIN's (*T*, ϵ) and OpenMax's (tail size, α) are selected on calibration data only, per seed. Bold indicates the results of the proposed PEOS-Net.

Averaged across seeds, PEOS-Net outperforms all three baselines on every open-set metric. AUROC reaches 0.8695 ± 0.0202, ahead of the strongest baseline, OpenMax (0.8384 ± 0.0129), by 3.1 percentage points; a similar margin holds for AUPR (0.8723 vs. 0.8536 for ODIN, the strongest baseline on that metric). The largest and most practically relevant margin is on FPR@95%TPR, where PEOS-Net achieves 0.4853 ± 0.0645 against 0.6320–0.6430 for the three baselines—a reduction of roughly 15–18 percentage points in the false-rejection rate at a fixed high recall of known-class samples, the operating regime most relevant to deployment. On OSCR, which jointly captures closed- and open-set behavior, PEOS-Net again leads (0.8363 ± 0.0142 vs. 0.8160–0.8185 for the baselines).

Notably, all three baselines achieve *higher* closed-set accuracy than PEOS-Net (0.9650 ± 0.0038 vs. 0.9387 for PEOS-Net's headline result). PEOS-Net's advantage is therefore not attributable to superior closed-set classification; rather, PEOS-Net trades a modest amount of closed-set accuracy for substantially improved open-set rejection, consistent with its architectural goal of separating the classification decision (prototype branch) from the rejection decision (energy branch) rather than optimizing a single objective for both.

We note that an earlier evaluation of OpenMax, using hyperparameters not selected under the calibration-only protocol, produced an anomalously low AUROC (0.5178, indistinguishable from random) that we now attribute to inadequate hyperparameter selection rather than a genuine limitation of the method for this domain. Under the corrected protocol - with tail size and α selected on calibration data, and a *post-hoc* sanity check confirming that mean rejection scores are higher for unknown than known samples in every seed - OpenMax performs comparably to MSP and ODIN, in line with expectations from the broader OSR literature. All three baselines land within a tight band of one another (AUROC 0.829–0.838), which is reassuring: it indicates that PEOS-Net's advantage over them is not an artifact of any single baseline being poorly tuned, but a consistent margin across three independently implemented open-set scoring mechanisms. PEOS-Net's hybrid fusion of prototype distance and independently learned energy scores remains the strongest performer throughout, consistent with the complementary-branch analysis in Section 5.

### 5.10 Computational efficiency

To quantify PEOS-Net's lightweight-deployment claim, we profiled its parameter count and inference latency against a plain CBAM-augmented EfficientNet-B0 classifier with no prototype or energy branch (the same architecture underlying the MSP and ODIN baselines in Section 5), on identical hardware (NVIDIA Tesla P100, Kaggle). Results are summarized in Table 12.

**Table 12:** Computational cost of PEOS-Net vs. a plain CBAM+EfficientNet-B0 classifier with no prototype or energy branch, measured on identical hardware (NVIDIA Tesla P100, Kaggle).

Model	Total params	Trainable params	Latency (ms/image)
Plain baseline (CBAM only)	4,479,775	425,066	8.54
PEOS-Net (full)	4,966,055	908,522	9.02

Latency is measured at batch size 32, matching the training batch size (Table 3), averaged over 100 iterations after warm-up.

PEOS-Net's prototype and energy branches add approximately 483K trainable parameters (a 2.1 × increase over the plain classifier head) and increase measured inference latency by 5.6% (8.54–9.02 ms/image), while the frozen EfficientNet-B0 backbone—common to both models—dominates the total parameter count in each case. We report measured latency and parameter counts rather than a theoretical FLOPs count, as these more directly quantify practical deployment cost; the marginal overhead observed here supports our characterization of PEOS-Net's open-set recognition machinery as lightweight relative to the shared frozen backbone both models carry.

### 5.11 Limitations

A limitation of the present evaluation is the scope of the unknown-class distribution. Our open-set test partition draws unknown samples from only two disease classes (Mosaic virus, Yellow Leaf Curl), sourced from the same PlantVillage corpus, imaging protocol, and crop species as the known classes. This constitutes a within-dataset class-holdout evaluation: it tests whether PEOS-Net can reject disease categories absent from its training taxonomy, but does not test robustness to the broader distribution shifts a deployed system would encounter in the field–different crops, uncontrolled illumination and backgrounds, insect damage absent from PlantVillage, or non-leaf imagery. We did not evaluate a leave-class-out sweep across multiple choices of held-out disease classes, nor an external, field-condition OOD source [e.g., PlantDoc (Singh et al., 2020)], and consequently cannot rule out that the reported open-set metrics are specific to the particular choice of Mosaic virus and Yellow Leaf Curl as the unknown classes. We treat these two experiments as important directions for future validation (discussed further in the Conclusion) rather than claims established by the present study.

## 6 Conclusion

This paper presented Prototype-Energy Open-Set Network (PEOS-Net), a training-efficient framework for open-set tomato leaf disease recognition. The architecture integrates a CBAM-enhanced, frozen EfficientNet-B0 backbone with two

complementary branches: an EMA prototype-memory branch for interpretable distance-based classification, and a parameter-distinct classifier head, sharing the same backbone, producing an energy-based uncertainty score, combined through a weighted hybrid fusion strategy. On an eight-class subset of the PlantVillage tomato dataset, PEOS-Net achieves a closed-set classification accuracy of 93.87%, an AUROC of 0.8711, and an OSCR of 0.8394, evaluated under a strictly separated calibration/final-test protocol in which normalization statistics, the fusion weight, and the rejection threshold are all fixed on a held-out calibration set before the final test partition is scored exactly once. Ablation experiments confirm that hybrid fusion consistently outperforms either branch individually, and paired statistical tests verify that the two branches capture complementary information—supporting the architectural design and addressing the redundancy observed in an earlier single-branch implementation. This evaluation is conducted within a single dataset source, with unknown samples drawn from two held-out PlantVillage disease classes under the same imaging conditions as the known classes (Section 5.11); we therefore present these results as a controlled open-set recognition study rather than a validated field deployment. Future work will focus on selective fine-tuning of deeper backbone layers under stronger regularization, incorporating explicit metric-learning objectives (e.g., triplet or contrastive loss) to further improve embedding-space separability, extending the present calibration protocol to a leave-class-out evaluation across a broader set of unknown disease categories to probe sensitivity to the specific choice of unknown classes, and evaluating against a genuinely external, field-condition OOD source (e.g., PlantDoc) to assess robustness beyond the PlantVillage distribution. Overall, PEOS-Net demonstrates that combining prototype-based geometric representations with an independent energy-based uncertainty estimator provides an effective, interpretable foundation for open-set diagnostic systems, motivating further validation toward field-deployed precision agriculture applications.

## Data Availability

The dataset used in this study is publicly available on Kaggle at: https://www.kaggle.com/datasets/kaustubhb999/tomatoleaf, released under a CC0: Public Domain license.
